# Function, Innervation, and Neurotransmitter Signaling in Mice Lacking Type-II Taste Cells

**DOI:** 10.1523/ENEURO.0339-19.2020

**Published:** 2020-01-30

**Authors:** Eric D. Larson, Aurelie Vandenbeuch, Catherine B. Anderson, Sue C. Kinnamon

**Affiliations:** 1 Department of Otolaryngology, University of Colorado Anschutz Medical Center, Aurora, CO 80045; 2 Rocky Mountain Taste and Smell Center, Aurora, CO 80045

**Keywords:** ATP, innervation, Pou2f3, serotonin, Skn-1a, taste

## Abstract

The *Skn-1a* transcription factor (*Pou2f3*) is required for Type II taste cell differentiation in taste buds. Taste buds in *Skn-1a*^-/-^ mice lack Type II taste cells but have a concomitant expansion of Type III cells, providing an ideal model to determine the relative role of taste cell types in response specificity. We confirmed that chorda tympani responses to sweet, bitter, and umami stimuli were greatly reduced in the knock-outs (KOs) compared with wild-type (WT) littermates. *Skn-1a*^-/-^ mice also had reductions to NaCl that were partially amiloride-insensitive, suggesting that both Type II and Type III cells contribute to amiloride-insensitive salt detection in anterior tongue. We also confirmed that responses to sour stimuli are equivalent in the KOs, despite the large increase in the number of Type III taste cells. To examine their innervation, we crossed the *Htr3a*-GFP (5-HT_3A_-GFP) reporter mouse with the *Skn-1a*^-/-^ mice and examined geniculate ganglion neurons for GFP expression and responses to 5-HT. We found no change in the number of 5-HT_3A_-expressing neurons with KO of *Skn-1a*. Calcium imaging showed that only 5-HT_3A_-expressing neurons respond to exogenous 5-HT, while most neurons respond to ATP, similar to WT mice. Interestingly, despite loss of all Type II cells, the P2X3 antagonist AF353 blocked all chorda tympani responses. These data collectively raise questions pertaining the source of ATP signaling in the absence of Type II taste cells and whether the additional Type III cells are innervated by fibers that would have normally innervated Type II cells.

## Significance Statement

Despite numerous studies, the role of specific taste cell types in taste responsivity and their connectivity to gustatory nerves is incompletely understood. Here we show that in *Skn-1a* knock-out (KO) mice where only Type I and III cells exist within taste buds, the number of gustatory ganglion cells innervating Type III cells remains unchanged and these neurons exhibit normal responses to gustatory neurotransmitters. Further, we show that although ATP release from taste buds is undetectable in *Skn-1a* KO mice, ATP is still required to drive gustatory neural output via the chorda tympani nerve. The source of the required ATP is unclear and begs further study.

## Introduction

Taste signals generated by the interaction of molecules with receptors located on taste cells are transmitted to gustatory neurons to elicit a sensation of taste. Taste buds comprise three elongate taste cell types that are defined by molecular features and physiological properties (for review, see Roper and Chaudhari, 2017). Type I cells are generally considered to have primarily a support role, similar to glial cells in the nervous system. Type II cells express the G-protein-coupled receptors and downstream effectors for bitter, sweet, and umami taste stimuli. These cells when stimulated release ATP via the large conductance CALHM1 channels to activate P2X receptors on gustatory afferent fibers (Taruno et al., 2013). Type III cells respond to acids (sour stimuli) via an apically-located proton channel (Bushman et al., 2015; Ye et al., 2016) and when stimulated, release 5-HT (Huang et al., 2011, 2008) to activate the 5-HT_3A_ receptors on gustatory afferents (Larson et al., 2015). The cell bodies of these neurons form part of the geniculate ganglion (VIIth cranial nerve), petrosal ganglion (IXth cranial nerve) and nodose ganglion (Xth cranial nerve). While 5-HT contributes to only a portion of the nerve response to taste, ATP is required for transmission of all taste modalities, as purinergic receptor antagonism or knock-out (KO) eliminates all nerve response to taste (Finger et al., 2005; Vandenbeuch et al., 2015). However, the role of ATP in the taste response for non-Type II cell mediated modalities remains elusive as release of ATP has only been detected from Type II cells (Huang et al., 2007; Romanov et al., 2007; Murata et al., 2010).

The development of Type II taste cells requires the transcription factor Skn-1a (*Pou2f3*). Skn-1a is a transcription factor (POU domain gene) involved in the regulation of gene expression in epidermal keratinocytes (Andersen et al., 1997) and regulates the generation and differentiation of taste cells (Matsumoto et al., 2011). Mice lacking Skn-1a do not express Type II taste cells and show deficient chorda tympani and glossopharyngeal nerve responses to sweet, umami, and bitter stimuli, but interestingly, although the number of Type III cells is increased by approximately 30% in taste buds of *Skn-1a*^-/-^ mice, acid responses are similar to those observed in wild-type (WT) animals (Matsumoto et al., 2011). Recently, Maeda et al. (2017) showed that the absence of Type II taste cells and the overexpression of Type III taste cells in the *Skn-1a*^-/-^ mice do not alter the number of nodose/petrosal ganglion cells that innervate Type III cells using retrograde transfer of wheat germ agglutinin (WGA). These results suggest that connections between taste cells and ganglion cells are not influenced by the type of cells expressed in the tongue but are predetermined. What is not clear, however, is whether the additional Type III cells in the *Skn-1a*^-/-^ mice are innervated by ganglion neurons expressing the serotonin receptor 5-HT_3A_, as in WT mice (Stratford et al., 2017). Here, we have crossed the *Skn-1a*^-/-^ animals with mice expressing GFP from the *Htr3a* promoter and examined the progeny for 5-HT_3A_ expression and function in the geniculate ganglion. Further, we examined the dependence of taste signaling on ATP in the *Skn-1a*^-/-^ mice that lack Type II taste cells.

## Materials and Methods

### Animals

Experiments were performed on *Skn-1a*^-/-^ and WT mice obtained from Ichiro Matsumoto at Monell Chemical Senses Center. Mice were crossed for several generation with C57Bl/6. 5HT3A-GFP mice were purchased from MMRRC (catalog #000273-UNC) and crossed with *Skn-1a*^-/-^ mice. Both males and females aged between four and six months were used. Mice were housed at the author’s institution in ventilated cages with access to water and food *ad libitum*. All experimental procedures were conducted in accordance with the Animal Care and Use Committee at the University of Colorado Anschutz Medical Center.

### RT-PCR

Separate pools of circumvallate and fungiform taste tissues were isolated from three *Skn-1a*^-/-^ and WT littermates. RNA was extracted in a QIAcube according to manufacturer’s instructions using reagents from QIAGEN. A 30-min DNase I treatment at room temperature to remove genomic DNA was included. Reverse transcription was performed using the QuantiTect Reverse Transcription kit which included an additional step for removal of genomic DNA. Two microliters of cDNA were added to the PCR reaction (Taq PCR Core). PCR conditions included an initial 5 min denaturation step followed by 40 cycles of 30-s denaturation at 95°C, 30-s annealing at 60°C and 45-s extension at 72°C and a final 7-min extension step. PCR primer sequences can be found in Table 1. We included fungiform cDNA from WT mice as a positive control and a no template negative control where water was added in place of cDNA. Amplified sequences were visualized by gel electrophoresis in 2% agarose gels stained with GelRed (Biotium).

**Table 1.** PCR primers

Gene	Accession number	Primer sequence (5′ to 3’)	Product size
*Calhm1*	NM_001081271	F: GTGCTTTCTCTGTGCCTTCT R: CGTACCACGAACGCTAGTAATG	240
*Skn1a* (*Pou2f3*)	NM_011139	F: GGCGATGGGAAAGCTGTAT R: CTCCAAAGTCAGGCGTATGT	249
Gustducin (*Gnat3*)	NM_001081143	F: GCAACCACCTCCATTGTTCT R: AGAAGAGCCCACAGTCTTTGAG	285
Snap25	NM_001355254	F: GGCAATAATCAGGATGGAGTAG R: AGATTTAACCACTTCCCAGCA	307

### Taste bud immunohistochemistry

Intraperitoneal injections of 5-hydroxy-L-tryptophan (5-HTP; 50 mg/kg; Sigma Aldrich) were performed on animals to increase 5-HT levels. After 1 h, mice were deeply anesthetized with urethane and transcardially perfused using 4% paraformaldehyde. For lingual tissues, after 4 h postfix and cryoprotection in 20% sucrose overnight, 12- to 16-μm cryostat sections were cut and mounted onto slides. After buffer washes with PBS, specific primary antibodies for each cell type were applied to the sections and immunoreacted overnight at 4°C. Secondary antibodies were reacted for 2 h at room temperature and mounted with Fluoromount (Southern Biotech). Z-stack images were collected on a Leica SP5 or SP8 confocal microscope. Antibodies and their sources are listed in Table 2.

**Table 2.** List of primary and secondary antisera

Antiserum	Company	Catalog number	Dilution	RRID
Chicken polyclonal anti-GFP	Aves Lab	GFP-1020	1:2000	AB_10000240
Rabbit polyclonal anti-PLCβ2	Santa Cruz Biotechnology	SC-206	1:1000	AB_632197
Goat polyclonal anti- GNAT3	Aviva Systems Bio	NC9510598	1:500	AB_10882823
Rabbit polyclonal anti-P2X3	Alomone	APR-016	1:500	AB_2313760
Goat polyclonal anti-SNAP25	Genetex	GTX89577	1:200	AB_10724125
Donkey anti-chicken 488	Jackson ImmunoResearch	703-546-155	1:1000	AB_2340375
Donkey anti-rabbit 568	Invitrogen	A10042	1:1000	AB_2534017
Donkey anti-goat 647	Invitrogen	A1157	1:1000	AB_2758603
DAPI	Invitrogen	62248	1:10,000	AB_2307445

### Geniculate ganglion immunohistochemistry

For geniculate ganglion imaging, geniculate ganglia were removed from paraformaldehyde-perfused heads and postfixed for 1 h. Ganglia were optically cleared with PACT (Cronan et al., 2015) and labeled with primary antibodies against GFP, P2X3, and SNAP25 and imaged on a Leica SP8 confocal microscope.

### Geniculate ganglion image quantification

Cleared whole mount ganglia were imaged in a single field of view with 1 μm axial spacing. Maximal z-projections of 10-μm substacks were created with ImageJ. Regions of interest (ROIs) were drawn around individual cells as identified by SNAP25 and DAPI and the average fluorescence value of each channel was measured for each ROI. Fluorescence intensity values were background subtracted and normalized for each image. Cells were scored as positive or negative for each channel using a threshold intensity value (Larson et al., 2015). Thresholds for each channel were determined by fitting intensity value histograms with Gaussian functions (two peak if bimodal). For bimodal distributions, threshold was determined by any value >2 SD above the median of the lower peak.

### Taste receptor cell quantification

Type III cells were counted from images of lingual slices labeled with antibodies against SNAP25. We chose SNAP25 to identify Type III cells as it has been previously shown as the broadest marker of Type III cells in the anterior tongue (Wilson et al., 2017). From each three-dimensional image collected, a plane from the middle, upper, and lower quadrants was extracted, and cell profiles with a clear nuclear region were counted per taste bud. This method minimized counting cells that persisted through multiple optical sections. Counts were parsed and plotted using a custom “R” script. Count data were analyzed using a Kruskal–Wallis test followed by Dunn test for multiple comparisons in R with the *dunn.test* and *FSA* packages (Dinno, 2017; Ogle et al., 2019).

### Taste bud innervation quantification

Image stacks of different taste fields were analyzed using ImageJ. Stacks were processed using “Subtract Background” (rolling ball radius 50 px), “Median” (radius 2), and “Auto Threshold” (Otsu method, stack histogram) to create multichannel binary images. ROIs were drawn around individual taste buds and the area, mean fluorescence, integrated density, and voxel size/volume were measured for each optical section. Using a custom R script, the total analyzed volume and the total labeled volume were calculated for each ROI. Innervation density was plotted as labeled volume/total volume.

### 5-HT_3A_-GFP, P2X3 nerve fiber quantification

Lingual sections were labeled with antibodies against GFP and P2X3. High-resolution 3D images were acquired on a Leica SP8 of all taste fields. Images were subject to a custom analysis pipeline to quantify the proportion of P2X3 immunoreactivity that overlapped with GFP immunoreactivity. In ImageJ, ROIs pertaining to individual taste buds were extracted and saved as new images for further processing which included Subtract Background (rolling ball radius 50 px), Median (radius 2), and Auto Threshold (Otsu method, stack histogram) to create multichannel binary images. Images and image metadata were imported to R using *raster*, *rgdal*, and *sp* packages (Pebesma and Bivand, 2005; Bivand et al., 2013, 2019; Hijmans, 2019). A custom script was used to calculate the volume of each taste bud ROI that was occupied by a P2X3+ and/or GFP+ voxel. Data are displayed as GFP:P2X3+ volume divided by P2X3+ volume using *ggplot2*. Statistical comparison was performed using a nonparametric Kruskal–Wallis test followed by Dunn test for multiple comparisons.

### Calcium imaging

Calcium imaging of isolated geniculate ganglion neurons was performed as previously described (Larson et al., 2015). Briefly, geniculate ganglia were extracted from euthanized animals, washed briefly in minimum essential medium with Earle’s balanced salts (MEM/EBSS; HyClone) and placed in MEM/EBSS supplemented with 1.25 mg/ml trypsin (Sigma-Aldrich) and 2.5 mg/ml collagenase A (Roche Diagnostics) for 30 min. Ganglia were washed with MEM/EBSS, gently triturated with a fire-polished glass pipette, and resuspended in HEPES buffer (136 mM NaCl, 5.6 mM KCl, 1 mM MgCl_2_, 2.2 mM CaCl_2_, 11 mM glucose, and 10 mM HEPES; pH 7.4). Cells were plated on poly-D-lysine (0.02 mg/ml) coated coverslips before loading with 2 μM fura-2 AM (Invitrogen) with 0.01% Pluronic F-127 (Invitrogen). Cells were continually perfused with HEPES buffer or HEPES buffer plus 10 μM ATP, 10 μM 5-HT, or HEPES buffer with 55 mM KCl. Cells were imaged using an inverted microscope equipped with a 40x oil immersion lens, Lamba 10–3 filter wheel (with 340 and 380 excitation filters; Sutter), and a QImaging Retiga R3 CCD camera. Acquisition was controlled using Imaging Workbench 6.1 (Indec Biosystems). Baseline emission ratio (340_ex_ and 380_ex_) values were determined as the first 10–15 s of recording and were subtracted from the peak ratio during stimulus perfusion. In the absence of a visible response, the value was taken 30 s after onset of stimulus perfusion. Data are plotted as baseline subtracted values divided by the peak values.

### Nerve recording

*Skn-1a*^-/-^ and WT littermates were anesthetized with an intraperitoneal injection of urethane (2 mg/kg; Sigma Aldrich) and placed in a head holder. The trachea was cannulated to facilitate breathing. Using a ventral approach, the chorda tympani was exposed, cut near the tympanic bulla and placed on a platinum-iridium wire. A reference electrode was placed in a nearby muscle. The signal was fed to an amplifier (P511; Grass Instruments), integrated and recorded using AcqKnowledge software (Biopac). The anterior part of the tongue containing the fungiform papillae was continuously stimulated with NH_4_Cl 100 mM, sucrose 500 mM, quinine 10 mM, MSG 100 mM, MSG 100 mM + IMP 0.5 mM, HCl 10 mM, citric acid 10 mM, NaCl (30, 100, and 300 mM). In some experiments, amiloride (100 μM), an epithelial Na^+^ channel (ENaC) blocker was added to NaCl. In some experiments, the P2X3 antagonist, AF353, was diluted in water (1 mM) and applied on the tongue for 10 min. Each stimulus was applied for 30 s and rinsed with water for 40 s. Since all responses could potentially be affected in the KO mice, each response was normalized to the baseline (Vandenbeuch et al., 2013, 2015; Larson et al., 2015). Although we drew similar conclusions when data were normalized to NH_4_Cl responses (data not shown), we present data as baseline-normalized as the cell type involved in the NH_4_Cl response is likely to be Type III cells (Oka et al., 2013). To analyze the data, the baseline integrated response was averaged over 10 s prior to stimulation of each tastant and subtracted from the average amplitude of each integrated response (over 30 s). The baseline subtracted responses were then divided by the respective baseline.

### ATP release

An enzymatic cocktail containing Dispase II (3 mg/ml; Roche) and Elastase (2.5 mg/ml; Worthington) diluted in Tyrode’s was injected under the circumvallate papillae. The epithelium was peeled after 20 min incubation in Tyrode’s and placed on a modified Ussing chamber (42 μl). The basolateral part of the papilla was bathed in Tyrode’s while the apical part was stimulated with 5 μl of artificial saliva, citric acid (20 mM), or NaCl (500 mM) diluted in artificial saliva. Only one stimulus was applied on each preparation and could not be rinsed because of the high sensitivity to mechanical stimulation. The Tyrode’s containing the releasate from taste buds was collected after each stimulation, transferred to a 96-well plate and placed in a plate reader (Synergy HT, Biotek). 42 μl of luciferase (ATP bioluminescence kit HS II; Roche) was automatically injected into each well and the luminescence reading was performed. Known concentrations of ATP solutions were also read to obtain a standard ATP curve and convert relative light units into ATP concentrations (nM). The Tyrode’s solution contained the following: 140 mM NaCl, 5 mM KCl, 4 mM CaCl_2_, 1 mM MgCl_2_, 10 mM glucose, 1 mM Na-Pyruvate, and 10 mM HEPES, pH adjusted to 7.4 with NaOH. Artificial saliva contained the following: 2 mM NaCl, 5 mM KCl, 3 mM NaHCO_3_, 3 mM KHCO_3_, 1.8 mM HCl, 0.25 mM CaCl_2_, 0.25 mM MgCl_2_, 0.12 mM K_2_HPO_4_, and 0.12 mM KH_2_PO_4_.

### Statistics

All statistical calculations were performed in Sigmaplot (V12.5, Systat Software, SCR_003210). Statistical tests within each figure are described in Table 3; *p *<* *0.05 was considered significant.

**Table 3.** Statistical table

Figure	Data structure	Type of test	Sample size	Statistical data
Figure 2*B*	Two-factors (genotype and taste field); data are counts	Kruskal–Wallis test followed by Dunn test for multiple comparisons	WT mice: 6 mice CV: 110 buds FF: 19 buds SP: 15 buds KO mice: 6 mice CV: 125 buds FF: 16 buds SP: 17 buds	χ^2^(5, *N* = 302) = 219.74, *p* < 0.0001 CV_WT vs CV_KO: *p* < 0.0001 FF_WT vs FF_KO: *p* = 0.0395 SP_WT vs SP_KO: *p* = 0.00331
Figure 3*A*	Two-factors (genotype and tastants); data are continuous	Two-way ANOVA	WT mice: *n* = 6 *Skn-1a^-/-^* mice: *n* = 6	Genotype: *F*_(1,91)_ = 12.755, *p* < 0.001 Interaction: *F*_(7,91)_ = 3.315, *p* = 0.004 Holm–Sidak’s multiple comparisons test: KO/WT, NH_4_CL: *p* = 0.282 KO/WT, Suc: *p* = 0.008 KO/WT, Qui: *p* = 0.034 KO/WT, Msg: *p* = 0.039 KO/WT, NaCl: *p* = 0.008 KO/WT, Msg+Imp: *p* = 0.007 KO/WT, Hcl: *p* = 0.127 KO/WT, Ca: *p* = 0.975
Figure 3*B*	Two-factors (genotype and tastants)	Two-way ANOVA	WT mice: *n* = 5 KO mice: *n* = 9	Genotype: *F*_(1,41)_ = 6.726, *p* = 0.014 Interaction: *F*_(2,41)_ = 0.976, *p* = 0.386
Figure 4*C*	Categorical	χ^2^	WT cells: 3262 from 3 mice KO cells: 4236 from 4 mice	χ^2^(2, *N* = 7498) = 5.312, *p* = 0.070
Figure 4*D*	Categorical	χ^2^	WT cells: 3262 from 3 mice KO cells: 4236 from 4 mice	χ^2^(3, *N* = 7498) = 5.064, *p* = 0.167
Figure 5*C*	Two-factors (genotype and stimulus)	Two-way ANOVA	WT cells: 7 KO cells: 6	Genotype: *F*_(1,38)_ = 0.0670, *p* = 0.797 Interaction: *F*_(2,38)_ = 0.0272, *p* = 0.973
Figure 5*D*	Two-factors (genotype and stimulus)	Two-way ANVOA	WT cells: 4 KO cells: 6	Genotype *F*_(1,29)_ = 0.0156, *p* = 0.902 Interaction: *F*_(2,29)_ = 0.409, *p* = 0.669
Figure 6*C*	Two-factors (genotype and taste field)	Kruskal–Wallis test	WT taste buds: 166 KO taste buds: 207	χ^2^(7, *N* = 302) = 7.0057, *p* = 0.4283
Figure 7*C*	Two-factors (genotype and taste field)	Kruskal–Wallis test followed by Dunn test for multiple comparisons	WT taste buds: 74 KO taste buds: 154	χ^2^(7, *N* = 302) = 57.047, *p* < 0.0001 CV_WT vs CV_KO: *p* = 0.0181 FOL_CV vs FOL_KO: *p* < 0.0135 FF_WT vs FF_KO: *p* > 0.05 SP_WT vs SP_KO: *p* > 0.05
Figure 9	Normal distribution	Paired *t* test vs artificial saliva	WT NaCl: 10 WT CA: 9 KO NaCl: 5 KO CA: 8	WT NaCl: *t*_(9)_ = 2.363, *p* = 0.0424 WT CA: *t*_(8)_ = 2.697, *p* = 0.027 KO NaCl: *t*_(4)_ = 1.262, *p* = 0.275 KO CA: *t*_(7)_ = 0.473, *p* = 0.650

## Results

### *Skn-1a*^-/-^ mice do not express canonical Type II taste cell markers

**Figure 1.**
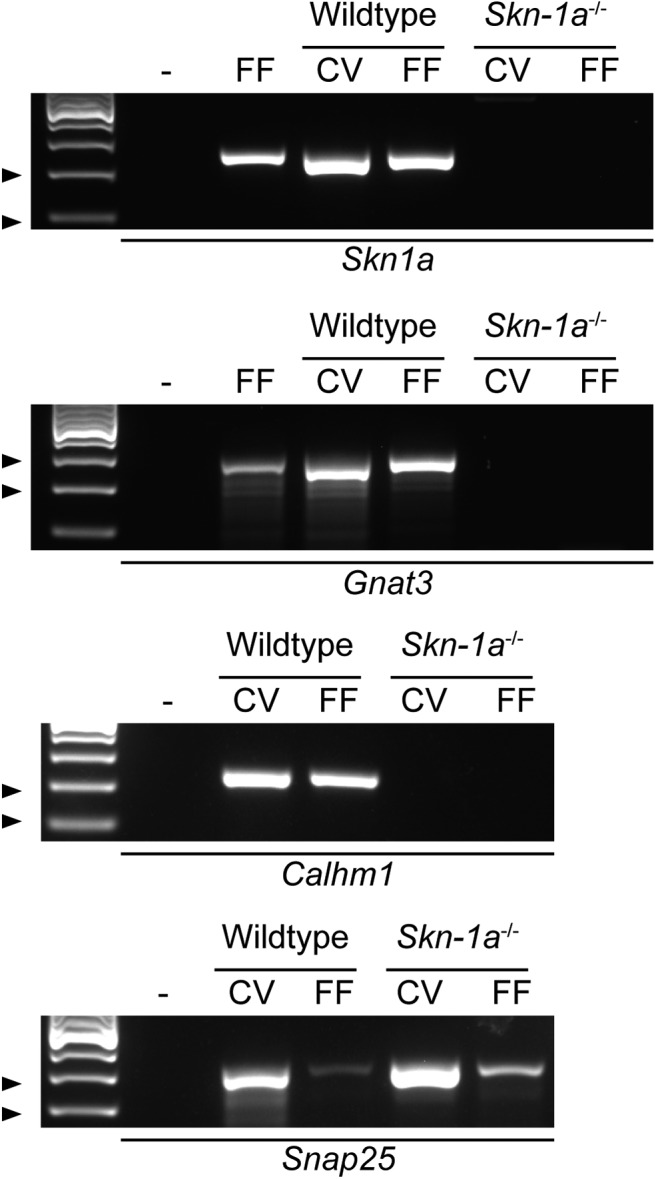
RT-PCR confirms lack of Type II cell markers. RNA was extracted from fungiform and circumvallate taste buds of WT and Skn-1a^-/-^ mice. After reverse transcription, cDNA was interrogated for the presence of *Skn1a*, *Gnat3*, *Calhm1*, and *Snap25.* Arrowheads denote ladder bands: *Skn1a*, 200 and 100 bp; *Gnat3*, 300 and 200 bp; *Calhm1*, 200 and 100 bp; *Snap25*, 300 and 200 bp. Data are representative of RNA extracted from three mice of each genotype; – is no template negative control, FF lanes in *Skn1a* and *Gnat3* gel are RNA from C57bl/6j fungiform taste buds.

**Figure 2.**
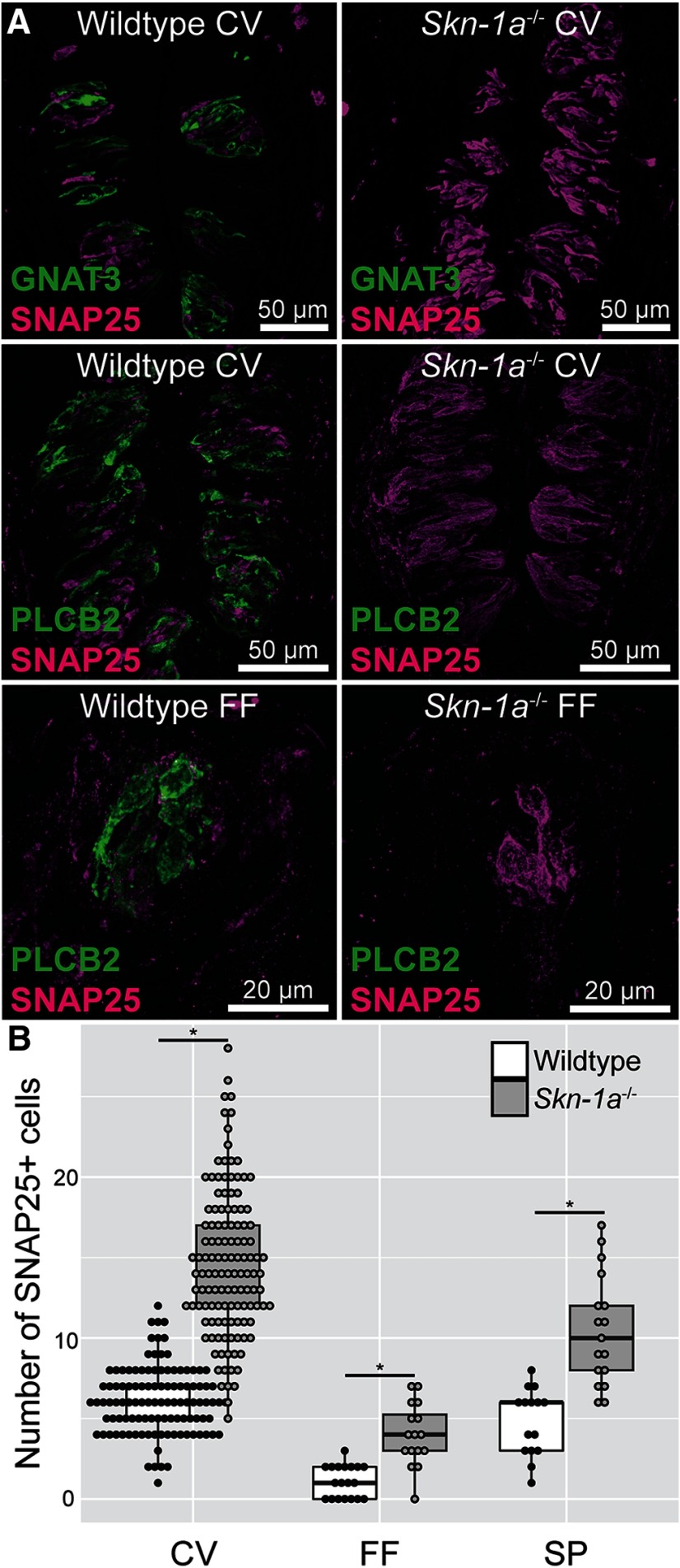
IHC confirms lack of GNAT3- and PLCβ2-expressing Type II cells in *Skn-1a^-/-^* mice. ***A***, Lingual sections from *Skn-1a^-/-^* and WT littermates were labeled with antibodies against SNAP25 (magenta) and GNAT3 (green) or PLCβ2 (green). In *Skn-1a^-/-^*, there was no detectable levels of Type II cell markers GNAT3 or PLCβ2. ***B***, Semi-quantitative IHC reveals increased numbers of Type III cells in circumvallate, fungiform, and soft palate taste buds. Each dot represents a single taste bud. Box plots are summaries of all datapoints. Acquisition settings were equalized between WT and KO for all channels. Images are maximal z-projections of 12- to 16-μm image stacks. CV = circumvallate, FF = fungiform; *p < 0.05 by Kruskal–Wallis test followed by Dunn test for multiple comparisons.

RT-PCR confirmed that fungiform and circumvallate taste buds of *Skn-1a*^-/-^ mice do not express *Pou2f3* (Skn-1a) or the Type II cell marker (*Gnat3*), however all taste buds expressed the Type III cell marker *Snap25* (Fig. 1). *Skn-1a^-/-^* mice do not express the ATP release channel *Calhm1*. We also used immunohistochemistry to confirm that no Type II cell markers were present in *Skn-1a*^-/-^ mice. As shown in Figure 2, neither GNAT3 nor PLCβ2 immunoreactivity was observed in *Skn-1a*^-/-^ mice. However, the number of Type III cells labeled with 5-HT and SNAP25 is increased in *Skn-1a*^-/-^ compared with WT as observed previously in Matsumoto et al. (2011). Cell counting of SNAP25 immunoreactive cell profiles confirms previous reports that the number of Type III cells is increased in CV taste buds. Additionally, we show that there is a significant, commensurate increase in Type III cells in the anterior taste fields as well (Fig. 2*B*). In mice pre-injected with the 5-HT precursor 5-HTP, the majority of Type III cells in *Skn-1a*^-/-^ mice were immunoreactive for 5-HT (data not shown).

### *Skn-1a*^-/-^ mice have diminished taste responses conveyed by Type II cells

**Figure 3.**
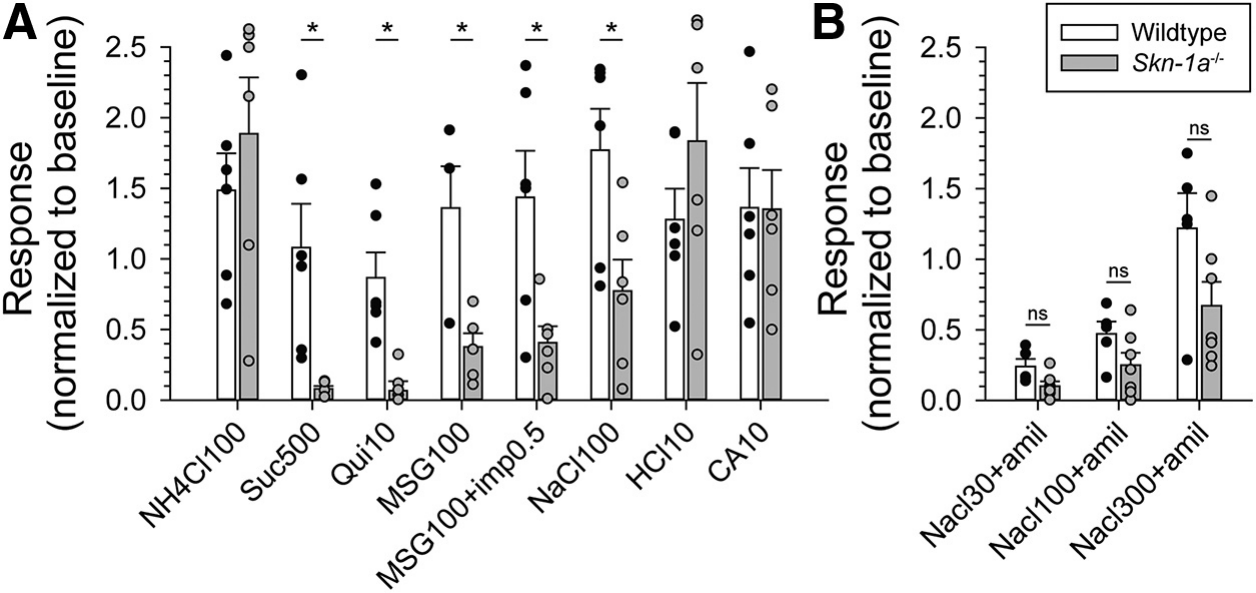
*Skn-1a^-/-^* mice have suppressed responses to Type II-mediated taste modalities. ***A***, Chorda tympani nerve activity of *Skn-1a^-/-^* and WT littermates was monitored in response to lingually applied taste solutions (100 mM NH_4_Cl, 500 mM sucrose, 10 mM quinine-HCl, 100 mM mono-sodium glutamate, 100 mM mono-sodium glutamate plus 0.5 mM inosine monophosphate, 100 mM NaCl, 10 mM HCl, and 10 mM citric acid). Integrated nerve activity over 30 s of stimulation was normalized to baseline; *N* = 6 mice for each genotype. ***B***, Baseline normalized chorda tympani activity of *Skn-1a^-/-^* mice in response to NaCl (30, 100, and 300 mM) with pre- and concurrent application of 100 μM amiloride. No significant differences were detected between WT and *Skn-1a^-/-^.* Bar charts indicate sample mean, error bars represent SEM, and superimposed dot plot represents each individual animal. ns = no significance; *p < 0.05 by two-way repeated measures ANOVA with Holm–Sidak *post hoc* test.

Chorda tympani responses to various tastants were compared between *Skn-1a*^-/-^ and WT (Fig. 3*A*). While responses to acids (citric acid and HCl) and NH_4_Cl were similar in both genotypes, responses to bitter (quinine), sweet (sucrose), umami (MSG with or without IMP), and salty (NaCl) stimuli were significantly decreased. NaCl responses were significantly decreased indicating that salt taste is also partially conveyed by Type II taste cells. When amiloride was added to NaCl at different concentrations, responses were diminished in the *Skn1a^-/-^* mice but not abolished suggesting that Type III cells participate in the transduction of the amiloride-insensitive salt response (Fig. 3*B*).

### *Skn-1a*^-/-^ mice have the same number of 5-HT_3A_-expressing geniculate ganglion neurons as the WT littermates

**Figure 4.**
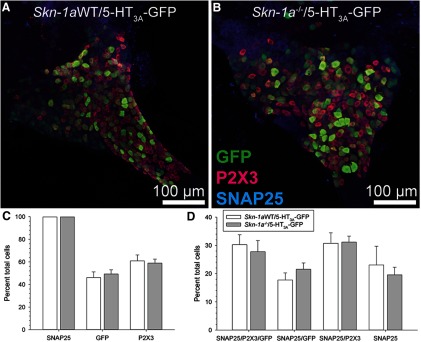
No change in the number of 5-HT_3A_-GFP geniculate ganglion neurons in *Skn-1a^-/-^* mice. *Skn-1a^-/-^* mice were crossed to 5-HT_3A_-GFP mice. ***A***, ***B***, Geniculate ganglia of *Skn-1a^-/-^/*5-HT_3A_-GFP and WT littermates (still expressing 5-HT_3A_-GFP) were labeled with antibodies against GFP (green), P2X3 (magenta), and SNAP25 (blue). The number of labeled cells was counted for each label. ***C***, Percent of total cells showing GFP, P2X3, or SNAP25 immunoreactivity. KO of Skn-1a had no effect. ***D***, Percent of total cells showing each combination of labels. KO of Skn-1a had no effect. Data are a summary of from three WT and four KO mice. Bar chart depicts sample mean and error bars show SEM.

Maeda et al. (2017) showed, via retrograde transfer of WGA from Type III cells, that the total number of nodose/petrosal ganglion neurons that innervate Type III cells remains unchanged in *Skn-1a*^-/-^ mice, despite the increased number of Type III cells. All gustatory neurons express the P2X-family purinergic receptors and a subset expresses 5-HT_3_ receptors which preferentially contacts Type III cells (Larson et al., 2015; Stratford et al., 2017). We tested the hypothesis that additional geniculate ganglia express 5-HT_3_ receptors to compensate for the additional Type III cells in *Skn-1a*^-/-^ mice, and that this change would be reflected when the 5-HT_3A_-GFP reporter mouse is crossed with *Skn-1a*^-/-^ mice. We showed that the total number of geniculate ganglion neurons expressing SNAP25, P2X3, or 5-HT3A was not significantly different between genotype (Fig. 4*C*). Additionally, we showed that the number of geniculate ganglion neurons expressing both P2X3 (gustatory neuron marker; Bo et al., 1999; Ishida et al., 2009; Dvoryanchikov et al., 2017) and 5-HT_3A_-driven GFP is similar to the number in WT littermates (Fig. 4*D*).

### *Skn-1a*^-/-^ mice show normal calcium response to ATP and 5-HT in geniculate ganglion neurons

**Figure 5.**
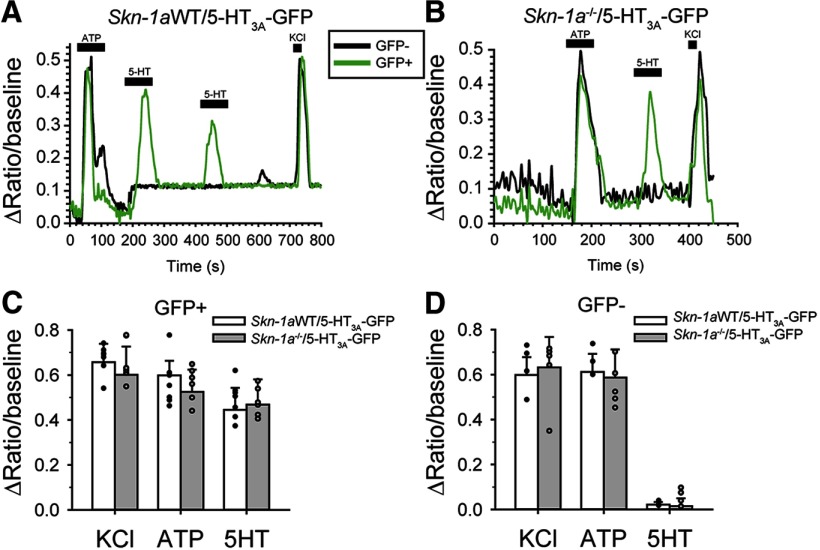
KO of Skn-1a does not affect responsiveness of geniculate ganglion neurons to ATP or 5-HT. *Skn-1a^-/-^* mice were crossed to 5-HT_3A_-GFP mice. ***A***, ***B***, Raw calcium imaging traces of isolated, individual GFP+ (green) or GFP- (black) geniculate ganglion neurons in response to 10 μM ATP, 10 μM 5-HT, or 55 mM KCl. ***C***, ***D***, Summary of responses to exogenous stimuli; *N* = 7 GFP+, 4 GFP- from 3 *Skn-1a^-/-^/*5-HT_3A_-GFP, and 6 GFP+ and 6 GFP- from 3 *Skn-1a*WT/5-HT_3A_-GFP. Bar charts indicate sample mean, error bars represent SEM, and superimposed dot plot represents each individual cell; *p <0.05 by Two-way ANOVA with Holm–Sidak *post hoc* test.

We next isolated geniculate ganglion neurons from *Skn-1a*^-/-^ mice crossed with 5-HT_3A_-GFP mice and examined response profiles to ATP and 5-HT. We observed similar response profiles in both KO and WT neurons. That is, GFP-expressing ganglion neurons responded to exogenous 5-HT and ATP while GFP-negative neurons responded only to ATP, similar to observations in WT neurons (Fig. 5*C*,*D*). Thus, in agreement with Maeda et al. (2017) we conclude that there is no change at the level of the geniculate ganglion to compensate for the increased number of Type III taste cells in *Skn-1a*^-/-^ mice. 5-HT signaling is not upregulated in *Skn-1a*^-/-^ mice.

### *Skn-1a*^-/-^ mice show normal innervation of taste buds

**Figure 6.**
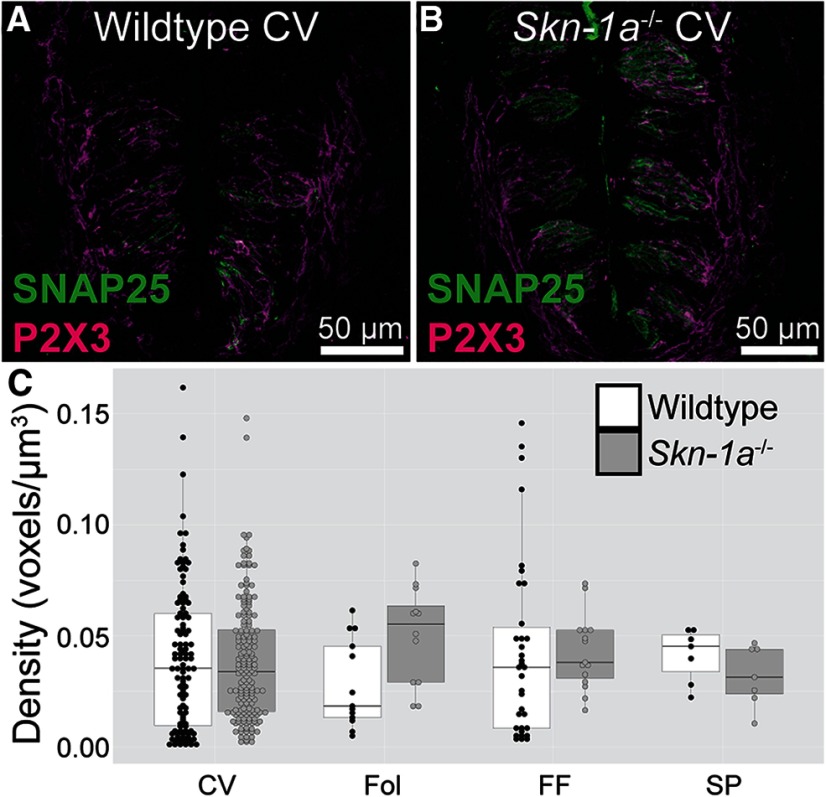
No change in the innervation density of taste buds in *Skn-1a^-/-^* mice. ***A***, ***B***, Lingual slices of *Skn-1a^-/-^* and WT littermates were labeled with antibodies against SNAP25 and P2X3. ***C***. P2X3 immunoreactivity density was calculated for each taste bud. Dots represent individual taste buds and the box plot demonstrates distribution of data. CV, circumvallate; Fol, foliate; FF, fungiform; SP, soft palate. Data are from six WT and five *Skn-1a^-/-^* mice. No significant interactions were found by Kruskal–Wallis test.

To determine if the taste buds of *Skn-1a*^-/-^ mice have similar numbers of nerve fibers compared with WT, we used an antibody against P2X3, since all gustatory nerve fibers express P2X3 (Bo et al., 1999; Ishida et al., 2009; Dvoryanchikov et al., 2017). The density of P2X3-labeled gustatory nerve fibers was compared in taste fields of KO and WT animals (Fig. 6). Total innervation density was calculated as percent labeled voxels divided by total number of voxels per imaged taste bud. No appreciable differences were observed between KO and WT in any taste field examined.

### 5-HT_3A_-GFP innervation of taste buds in *Skn-1a*^-/-^ mice

**Figure 7.**
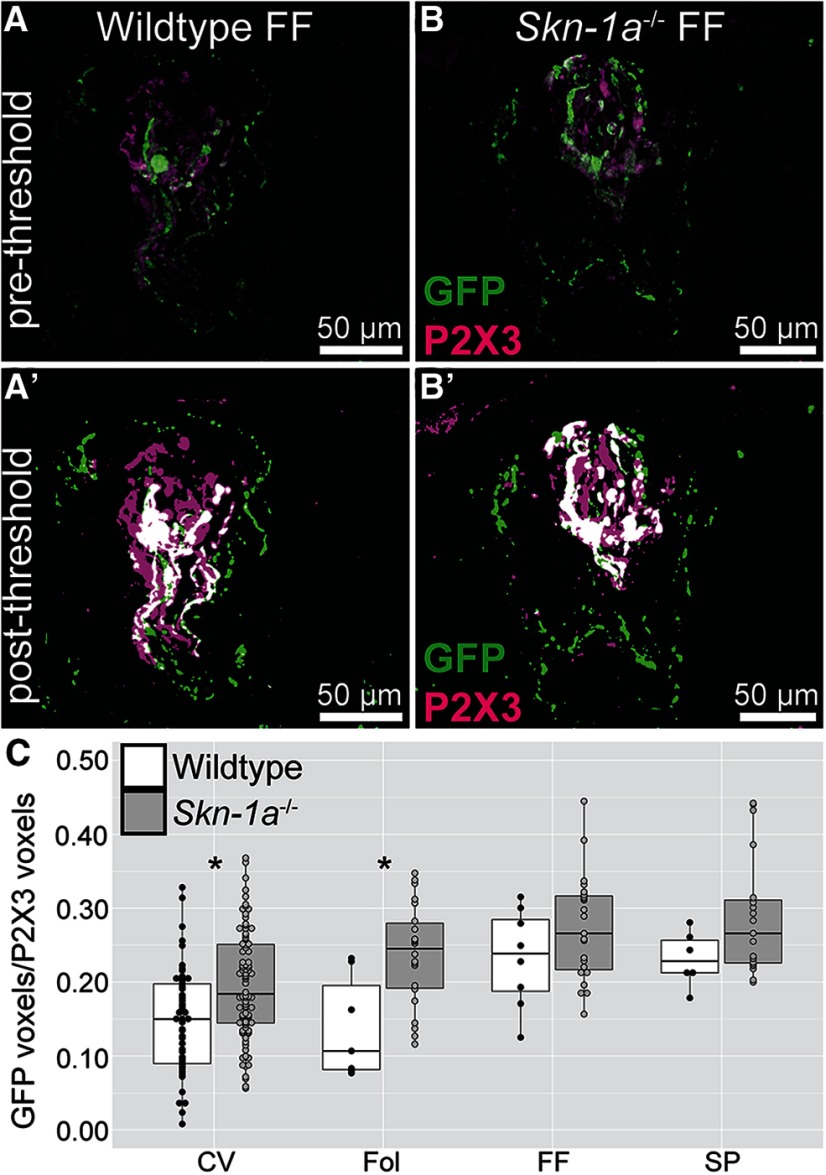
5-HT_3A_-GFP innervation in *Skn-1a^-/-^* mice. 5-HT_3A_-GFP mice were crossed with *Skn-1a^-/-^*. *Skn-1a^-/-^* mice that had the GFP transgene and WT littermates that also had the GFP transgene were used for experiments. ***A***, ***B***, Maximal z-projections of fungiform taste buds from WT and KO mice labeled with antibodies against GFP (green) and P2X3 (magenta). ***A’***, ***B’***, Same image as ***A***, ***B*** but after prepressing which included Otsu thresholding. ***C***, Analysis was performed on each optical plan of individual taste buds to quantify the relative immunoreactivity of P2X3 and GFP; *p < 0.05 by Kruskal–Wallis test followed by Dunn test for multiple comparisons. Three to four mice from each genotype were used, and 228 taste buds were quantified.

We have now shown that the overall level of P2X3+ innervation is unchanged and the overall number of 5-HT_3A_-GFP (Type III cell innervating) ganglion cells remains unchanged in *Skn-1a*^-/-^ mice. This raises the possibility that the increased Type III cells in *Skn-1a*^-/-^ mice are innervated by either (1) non 5-HT_3A_ neurons, (2) increased branches of 5-HT_3A_ neurons, or (3) no neurons at all. The most former seems unlikely, as if the new Type III taste cells were innervated by a new population of neurons, we would have expected to see increased chorda tympani responses to sour and salty as more fibers would be activated. In fact, we observed decreased salty responses and unchanged sour responses in *Skn-1a*^-/-^ mice. Thus, a more plausible explanation is that either the “new” Type III cells of *Skn-1a*^-/-^ mice are not innervated or are innervated by increased branching of neurons. To address this, we imaged taste buds of *Skn-1a*^-/-^ mice (and WT littermates) crossed with 5-HT_3A_-GFP mice. Quantitative analysis of these images showed a subtle increase in the proportion of P2X3-immunoreactive fibers that were also 5-HT_3A_-GFP+ when Skn-1a was KO. While significance was only achieved in more posterior taste fields, a positive trend was observed in the anterior fields (Fig. 7).

### *Skn-1a*^-/-^ mice require ATP to transmit gustatory neural responses

**Figure 8.**
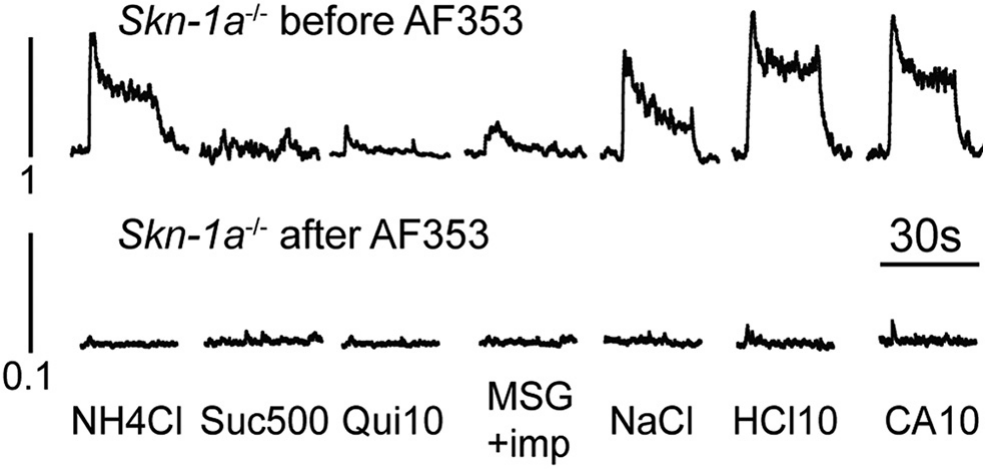
Purinergic receptor antagonism eliminates residual chorda tympani responses to lingually applied taste solutions. The chorda tympani response to taste solutions was measured in *Skn-1a^-/-^* before and after application of 1 mM AF353. Data are representative integrated responses to each stimulus. The *y*-axis scale is an order of magnitude smaller on the responses after AF353. Responses are representative of four animals.

ATP is required for the transmission of all taste qualities (Finger et al., 2005; Vandenbeuch et al., 2015) and the P2X3 antagonist AF353 blocks all taste responses (Vandenbeuch et al., 2015). When applying AF353 for 10 min on the anterior part of *Skn-1a*^-/-^ tongues, chorda tympani responses to all taste stimuli were totally abolished (Fig. 8). These results suggest that ATP is required to transmit the signal from taste cells to nerve fibers.

### *Skn-1a*^-/-^ mice do not release ATP to any tastants

**Figure 9.**
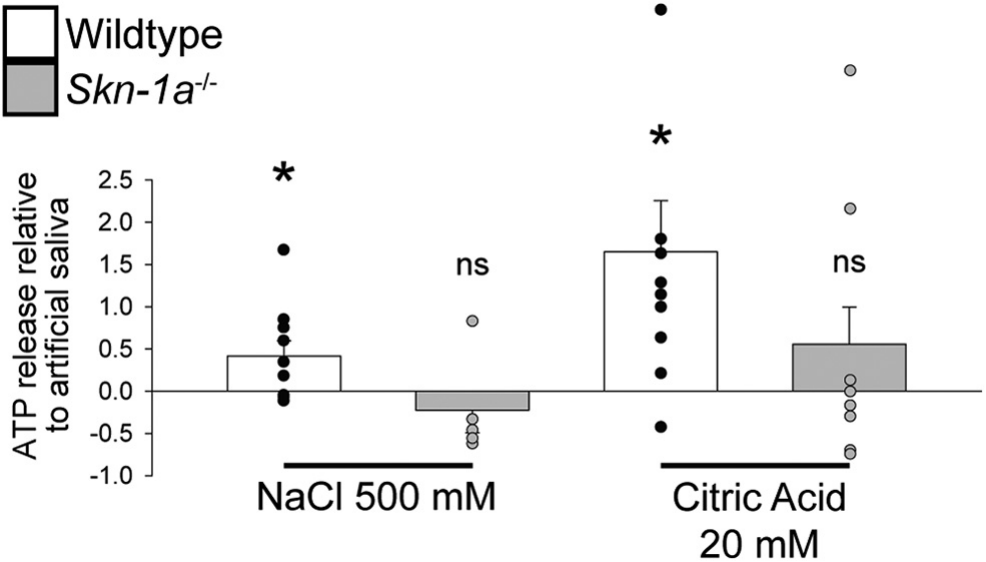
No detectable ATP release from taste buds of *Skn-1a^-/-^* mice. ATP release was measured from peeled circumvallate epithelium of *Skn-1a^-/-^* and WT littermates via luciferase assay. Stimuli were 500 mM NaCl, 20 mM citric acid, and artificial saliva. Data are presented relative to artificial saliva. Bar charts indicate sample mean relative to artificial saliva, error bars represent SEM, and superimposed dot plot represents each individual trial; *p < 0.05 compared with artificial saliva; ns = not significant compared with artificial saliva; *N* = 14 WT and *N* = 8 *Skn-1a^-/-^*mice.

To clarify the source of ATP required for the taste signal transmission in *Skn-1a*^-/-^ mice, the amount of ATP released to different tastants was measured. Using a luciferase assay, no ATP was released from taste buds of the *Skn-1a*^-/-^ mice following NaCl or citric acid stimulation. Conversely, ATP was released in the WT animals in response to the same stimuli (Fig. 9). Although we cannot rule out the possibility that the technique used was not sensitive enough, it seems unlikely that the source of ATP required for transmission comes from Type III cells in the *Skn-1a*^-/-^ mice.

## Discussion

In this manuscript we confirm earlier studies showing that the transcription factor Skn-1a is required for generation of Type II taste cells (Matsumoto et al., 2011) and that the resulting expansion of Type III cells in the *Skn-1a*^-/-^ mice does not alter the number of innervating gustatory neurons (Maeda et al., 2017). Here, we extend these earlier studies to show that despite the large increase in the number of serotonergic Type III taste cells in the *Skn-1a*^-/-^ mice, the ratio of geniculate ganglion neurons expressing the serotoninergic target receptor 5-HT_3A_ is unchanged with respect to WT mice. Further, we show that *Skn-1a*^-/-^ mice still require ATP for the transmission of sour and salty tastes to the nervous system despite the absence of Type II taste cells, which are the only taste cells known to release ATP.

We first confirmed using RT-PCR that the transcription factor *Skn-1a*, the G-protein *Gnat3*, and ATP release channel *Calhm1* are indeed missing in the *Skn-1a^-/-^* mice. In addition, we confirmed that the Type III cell marker *Snap25* is present in both WT and KO mice, ensuring the integrity of the mRNA in the KO mice. We then used immunohistochemistry to show that the *Skn-1a*^-/-^ mice lack expression of the canonical Type II taste cell marker PLCβ2 and that SNAP25 expressing Type III taste cells were expanded in both anterior and posterior taste fields to compensate for the loss of Type II taste cells. We also confirmed, using chorda tympani nerve recording of *Skn-1a*^-/-^ mice, that responses to stimuli normally transduced by Type II cells, i.e., bitter, sweet, and umami, were largely missing in the KOs compared with WT littermates. Also, in agreement with Matsumoto et al. (2011), we showed that responses to sour (acidic) stimuli were similar in KO and control mice, despite the large expansion of Type III cells. However, Matsumoto et al. (2011) showed a non-statistically significant decrease to NaCl, while we found responses to NaCl were significantly reduced, although not eliminated in the KOs, and that other sodium containing stimuli (e.g., MSG) showed small residual responses in the KO. We hypothesize that this difference is mainly due to experimental conditions: we used urethane anesthesia and baseline normalization while Matsumoto et al. (2011) used pentobarbital plus urethane anesthesia and NH_4_Cl normalization. Larson et al. (2015) have shown that sodium pentobarbital interferes with accurate measurement of NaCl responses. Our data suggest that Type II cells transduce a portion of the responses to NaCl, as others have suggested, although this point is still debated (Oka et al., 2013; Lewandowski et al., 2016; Roebber et al., 2019). Two distinct mechanisms are responsible for transduction of NaCl, an amiloride-sensitive mechanism, mediated by the epithelial Na channel ENaC and limited to anterior tongue (Kretz et al., 1999; Chandrashekar et al., 2010), and an unidentified amiloride-insensitive mechanism, found in all taste fields. Our results show that the addition of amiloride to NaCl does not completely abolish the salt response in the *Skn-1a^-/-^* mice suggesting that a portion of the amiloride-insensitive response is conveyed by Type III cells. Both Type II and Type III cells were previously thought to be responsible for amiloride-insensitive salt taste (Oka et al., 2013; Lewandowski et al., 2016). However, a recent study using Pirt-GCamp6 mice showed NaCl responses in fungiform taste buds were present only in Type II taste cells, and that these responses were entirely amiloride-insensitive (Roebber et al., 2019). These latter data are inconsistent with our data, which show a significant amiloride-insensitive response in mice lacking Type II taste cells. The amiloride-sensitive salt taste is believed to be transduced by a cell Type Independent of Type II and Type III cells in mice (Vandenbeuch et al., 2008; Chandrashekar et al., 2010), although whether it is a unique cell type or a subset of Type I cells is still unclear.

PKD1L3 is expressed in Type III cells of posterior tongue, and transgenic mice expressing WGA from the *Pkd1l3* promoter show WGA in Type III cells of circumvallate and foliate papillae as well as in petrosal ganglion neurons (Yamamoto et al., 2011). Maeda et al. (2017) crossed these PKD1L3-WGA expressing mice into the *Skn-1a*^-/-^ mice and examined the circumvallate taste buds and petrosal ganglion neurons for evidence of WGA protein. WGA protein was much higher in the taste buds of the KO mice than WT, but the number of ganglion cells containing WGA was not different from control mice, leading them to conclude that ganglion cell innervation is not regulated by taste cell type. We have expanded on this observation to examine the geniculate ganglion using mice expressing GFP from the serotonin receptor *Htr3a* promoter. Geniculate neurons expressing 5-HT_3A_-GFP preferentially innervate the serotonergic Type III taste cells and these neurons respond to both 5-HT and ATP, while neurons lacking GFP expression respond only to ATP (Larson et al., 2015; Stratford et al., 2017). A recent study confirms that most 5-HT_3A_ fibers innervate Type III cells. Secondary analysis of single cell RNA-sequencing data shows that geniculate ganglion neurons that respond to sour lingual stimuli are enriched with *Htr3a* (5-HT_3A_; data not shown; Dvoryanchikov et al., 2017; Zhang et al., 2019). We crossed the 5-HT_3A_-GFP mice into the *Skn-1a*^-/-^ mice and asked whether the ratio of GFP expressing neurons differed between KO and WT mice. Our results suggest that while the Type III taste cells of the KO are expanded, the 5-HT_3A_ expressing ganglion cells do not undergo a similar expansion, in general agreement with Maeda et al. (2017). Further, we showed that only the GFP-labeled ganglion neurons responded to exogenous 5-HT, while the majority of neurons responded to ATP, as with the WT animals. These results beg the question of whether the “extra” Type III cells in the KO mice are actually innervated. To assess this, we compared the innervation density of P2X3-expressing nerve fibers surrounding taste buds of circumvallate, foliate, fungiform, and palatal taste buds in KO and WT mice. All gustatory geniculate ganglion neurons express P2X3 (Bo et al., 1999; Ishida et al., 2009; Dvoryanchikov et al., 2017) and thus P2X3 is a reliable marker for taste bud innervation density. However, we found no difference in the overall innervation density between taste buds of the KO and WT mice, suggesting that all taste cells are likely to be innervated. It still remains possible that either 5-HT_3A_-expressing ganglion neurons have increased nerve fiber branching to innervate the expanded population of Type III cells in the KO, or the ganglion neurons expressing only P2X3 are now innervating the Type III cells. To address this, we quantified the proportion of P2X3-immunoreactive nerve fibers that were also 5-HT_3A_-GFP+ in *Skn-1a^-/-^* and *Skn-1aWT* mice crossed with 5HT3A-GFP mice (Fig. 7). We observed slight increases in the proportion of GFP+ gustatory fibers, although significance was only reached in the circumvallate and foliate papillae. This suggests that increased branching of GFP+ fibers could be occurring *Skn-1a^-/-^* mice. This would be consistent with observations that chorda tympani response to sour and salty stimuli are not increased, as one would expect much larger nerve signals if more neurons are being recruited from the increased number of Type III cells. However, further studies will be required to explore this question in more detail with enhanced resolution.

Previous studies have shown that ATP is required for the transmission of all taste qualities (Finger et al., 2005; Vandenbeuch et al., 2015), although only Type II cells are known to release ATP (Huang et al., 2007; Romanov et al., 2007, 2018; Taruno et al., 2013). The source of ATP required for transmission from Type III cells to gustatory afferents has remained an enigma, although it has been assumed to require the presence of Type II cells. Thus, it was of interest to examine the role of ATP in *Skn-1a*^-/-^ mice which lack Type II cells. Using a luciferin/luciferase assay, we found that taste buds of WT mice release detectible amounts of ATP to sour and salty stimuli applied to the apical membrane, but the *Skn-1a*^-/-^ mice failed to release ATP over background levels in the tissue. The finding that ATP release, presumably from Type II cells, is elicited by sour and salty was surprising and consistent with a model where signaling between taste receptor cells occurs (Huang et al., 2009). In this situation, activation of Type III cells by sour or salty stimuli causes indirect activation of Type II cells to release ATP. ATP release to sour and salty stimuli is specific to Type II cells as it is absent in *Skn-1a*^-/-^ mice.

We found that the purinergic receptor antagonist AF353 blocked transmission of sour and salty tastes in the KO as well as in the WT littermates. Thus, purinergic signaling is still required in these mice that lack Type II taste cells. But what is the source of the ATP, if not Type II cells? Our best explanation is that small amounts of ATP may be released from Type III cells that are undetectable by our assay and others’ attempts to measure ATP release from Type III cells (Huang et al., 2007; Romanov et al., 2007). One possibility is that ATP is co-released with serotonin (or other transmitters) from synaptic vesicles, thus it may be very focal and difficult to detect in this type of assay. Indeed, ATP is very commonly co-packaged in synaptic vesicles and often co-released with transmitters such as norepinephrine and acetylcholine [as reviewed in Borges (2013) and Burnstock (2006)]. Additionally, analysis of data from Qin et al., 2018 shows that using single cell RNA-sequencing *Slc17a9* (VNUT) is detectable in both Type II and III cells. While Iwatsuki et al. (2009) showed immunoreactivity to VNUT only in Type II cells the discrepancy could arise from the sensitivity of the assays used. These findings support the hypothesis that ATP could be released from vesicles of Type III cells at low levels sufficient for synaptic communication but insufficient for detection by conventional methods.

We also propose that pannexin channels could be a source of ATP release from Type III cells. While KO of pannexin-1 shows no phenotype, it is possible that in the absence of Type II cells and CALHM1 channels, pannexin-1 could take over a role for ATP release. Indeed, analysis of single cell RNA-sequencing data from Qin et al. (2018) shows detectable levels of *Panx1* in the majority of Type III cells (data not shown). A final possibility is that background levels of ATP in the tissues serve to keep gustatory afferents in a slightly depolarized state, so that release of serotonin or other transmitters will be sufficient to generate action potentials in the ganglion cells. Further studies will be required to resolve this issue.

## Synthesis

Reviewing Editor: Anna Menini, SISSA (International School of Advances Studies) Decisions are customarily a result of the Reviewing Editor and the peer reviewers coming together and discussing their recommendations until a consensus is reached. When revisions are invited, a fact-based synthesis statement explaining their decision and outlining what is needed to prepare a revision will be listed below. The following reviewer(s) agreed to reveal their identity: NONE.

This study presents interesting results for understanding the connectivity between taste cells and taste nerves. As the authors themselves state, this is a study that confirms and extends earlier findings from Matsumoto et al (2011) and Maeda et al (2017). The authors extend those earlier reports by showing that serotonergic geniculate ganglion neurons (those neurons that normally innervate Type III taste bud cells) do not increase in number or proportion in Skn-1a-/- mice despite the overrepresentation of Type III taste cells. They also report that taste stimulation of Skn-1a-/- mice elicits ATP release even in the absence of Type II taste bud cells, although it was not possible to detect measurable ATP release. The work is carried out carefully and the findings seem solid. However, authors should carefully address the specific concerns of each Reviewer listed below. Please note that Figure 5E is not acceptable as it seems to combine a control dataset of calcium imaging data in WT (5-HT-GFP) mice published in 2015 with a recently obtained dataset in KO mice. Please update the figure with calcium imaging data obtained from WT and KO littermates.

Reviewer 1

(1) A major difference between this work and the original report by Matsumoto et al (2011) is that in Skn-1a-/- mice, chorda tympani responses to NaCl were unaffected (Matsumoto et al) vs. significantly reduced (present report). This point is important because it bears upon the question of which cell(s) transduce salt taste. As the author(s) state, their findings here suggest that Type II taste cells are involved in salt taste (the findings of Matsumoto et al would argue the opposite). It is incumbent upon the present author(s) to determine/explain why their results differ from what has already been reported. Yet, no experiments or convincing explanation is provided.

(2) The summary of statistics (Table 3) is impressive. Some of the statistical information could be included in the figures and legends, too. Specifically, what are the error bars on the plots–s.d., s.e.m., 95CI? That should be stated in the legends. Also, in the figures themselves, the data points should be plotted instead of presenting only bar charts, especially when the sample sizes are small (n<10) as in many of these figures (c.f., Drummond GB and Vowler SL, 2011, “Show the data, don’t conceal them.” J Physiol 589: 1861-1863; Weissgerber et al, 2015, “Beyond bar and line graphs: time for a new data presentation paradigm.” PLoS biology 13.4: e1002128). Many top-line journals are adopting this standard.

The last entry in Table 3 states “Fig 7” but the data presumably refer to Fig. 8.

(3) In Figure 3, particularly in the absence of raw data traces, the author(s) could explain the y-axes better. Specifically, what is meant by “normalized to baseline”? The author(s) explanation in Methods refers readers to Larson et al (2015) and Vandenbeuch et al (2015), but isn’t the correct (original) citation Vandenbeuch et al 2013? And even there, this data “normalization” is a bit unclear. Raw data traces (such as in Figure 7) show a very small baseline with an evoked signal (e.g., mean over 30 sec) severalfold larger. If as the author(s) state the mean signal is “normalized” to the baseline, wouldn’t this yield values in the 10-100 fold range, not 0.1-2 fold as in the figures? Do the authors mean the baseline is “subtracted” from the averaged signal? If not, what exactly is the normalization and how is it achieved?

(4) In Figure 5, the ordinates on the raw signals of 340/380 emission data (Fig 5A,B) are unclear, for similar reasons as in point 3 above. In any case, wouldn’t a more accurate way to present Fura2 data be to calculate Δ[Ca2+] and if necessary, normalize those values? Also, in Fig 5F it seems a bit strange to use data from a 4-5 year old publication (Larson et al 2015) as controls in the present experiments. It’s not clear to this reviewer that this is an acceptable practice. Lastly, the author(s) should explain the brackets and numbers printed alongside the points in the plot.

(5) How the author(s) plot their data in Fig 6C is very innovative and imaginative. However, there is some confusion about what is plotted on the abscissae. More explanation of how to read these plots would help the reader a great deal.

(6) The argument that purinergic signaling is maintained in Skn-1a-/- mice, despite the inability to detect ATP release, hinges entirely on the finding that topically-applied AF353 (1 mM over 10 min) eliminates all taste-evoked chorda tympani responses in these mice. This discrepancy (no taste-evoked ATP release but taste-evoked purinergic signaling) is not resolved and the explanations proposed by the author(s) are not very satisfactory. Further, if Calhm1 channels are absent in Skn-1a-/- mice, do the author(s) have an explanation for the proposed ATP release mechanism?

(7) In the Discussion (line 242) the author(s) write that their “data suggest that Type II cells transduce a portion of the responses to NaCl, as others have suggested....”. They cite Lewandowski et al 2016 and Oka et al 2013. However, Roebber et al (2019), cited later for other reasons, should also be included here. Didn’t they also show that Type II taste cells are involved in salt transduction?"

Reviewer 2

(1) Quantitative data of taste bud cells in fungiform papillae in Skn1a KO mice is missing. It seems that Matsumoto et al. (2011) and Maeda et al. (2017) showed the expansion of type III cells only in circumvallate (and/or foliate) papillae because they used PKD1L3 as a type III cell marker that is not expressed in fungiform papillae. So, they did not show the quantitative data of type III cells in taste buds of fungiform papillae. Although the authors performed RT-PCR and immunostaining in the fungiform papillae, from the data they do not really indicate that the number of type III cells was increased and that type II cells were replaced by the expanded type III cells. Taste bud cells in soft palate are innervated by the neurons in the geniculate ganglion, so quantitative data in soft palate is also necessary.

(2) The authors used 5HT3A-GFP mice and showed that the number of GFP-positive neurons in Skn1a KO mice is equivalent with wild type mice. However, unlike the detection of the transsynaptic tracer, wheat germ agglutinin, from type III cells, unchanged population of 5HT3A-expressing neurons in taste ganglia does not mean the unchanged population of neurons innervating type III cells. All 5HT3A-positive cells receive signals from type III cells? All type III cells connect to only 5HT3A-positive cells? The result that the number of GFP-positive cells was similar between wild type and Skn1a KO mice suggests the possibilities: (1) P2X3+/5HT3A- neurons innervate expanded population of type III cells or (2) 5HT3A+ nerve branches are increased around taste buds. The authors examined P2X3-positive nerve terminals around taste buds and showed unchanged total innervation density of taste nerves between wild type and KO mice. Since GFP can be detected in nerve fibers around taste buds in 5HT3A-GFP mice (Stratford et al. 2017), similar experiments should be done using 5HT3A-GFP mice to determine whether 5HT3A-expressing neurons have expanded nerve branches in Skn1a KO.

(3) The authors showed the decreased nerve responses to NaCl in Skn1a KO mice, inconsistent with the previous data (Matsumoto et al. 2011). Any potential explanation? Could genetic background be a factor? Decreased nerve responses to NaCl in Skn1a KO mice and the results that response to NaCl with amiloride was similar between wild type and Skn1a KO mice suggest that amiloride-sensitive components to NaCl (i.e., ENaC-mediated salt taste) are involved in type II cells?

(4) The authors showed that ATP is released from circumvallate epithelium by the stimuli of NaCl and citric acid in wild type mice that have type II and type III cells, but not in Skn1a KO mice even though Skn1a KO mice have type III cells which mediate high salt and sour tastes. These results suggest that type II cells are activated directly or indirectly by the stimuli of NaCl and citric acid and then release ATP in wild type mice. The authors should address this possibility.

(5) The authors only showed that immunoreactivities to GNAT3 and PLCb2 were absent in Skn1a KO taste buds. But, from these histochemical data, it cannot be really claimed that no type II cells were present.

(6) Line 21-22. “...only type I and II cells exist within...” II should be III.
